# Study of the effects of nanoplastics ingestion in a freshwater fish (*Danio rerio*)

**DOI:** 10.1080/07853890.2021.1897423

**Published:** 2021-09-28

**Authors:** Simon Brand, Daniela Nunes, Rita Bastos, Jairo Falla, Mário S. Diniz

**Affiliations:** a Université de Lorraine, Laboratoire Interdisciplinaire des Environnements Continentaux (LIEC), IUT Thionville-Yutz, Espace Cormontaigne, Yutz, France; b Departamento de Ciências da Vida, Faculdade de Ciências e Tecnologia, Universidade NOVA de Lisboa, Caparica, Portugal; c UCIBIO, REQUIMTE, Departamento de Química, Faculdade de Ciências e Tecnologia, Universidade NOVA de Lisboa, Caparica, Portugal

## Abstract

**Introduction:**

The pollution by nanoplastics (NPs) has been shown in several ecosystems and aquatic biota, worldwide [1,2]. It is known that NPs can contaminate the food chain [1,2]. However, the toxicity of NPs in aquatic animals, especially freshwater fish, has been less studied [2]. Thus, this work intended to answer the question: how exposure to NPs affects the activities of antioxidant enzymes and the total antioxidant capacity in a freshwater fish species (*Danio rerio*). It also aimed to assess the vulnerability of this species to environmental contamination by NPs.

**Materials and methods:**

The fish (*D. rerio*), were randomly distributed by three aquaria of 15 L (*n* = 36; weight: 0.21 ± 0.06g; length: 2.7 ± 0.3 cm) and exposed during 7 and 14 days to different concentrations of NPs *via* food ingestion (fed daily). Thus, food pellets were previously embedded in a suspension of NPs (Sigma-Aldrich) containing 50 µg NPs/L and 100 µg NPs/L, respectively. Fish were sampled at the end of the exposure periods to assess oxidative stress biomarkers (antioxidant enzymes and TAC levels). In brief, samples (whole fish) were homogenised in a buffer solution (PBS), centrifuged at 10,000 × *g* (15 min at 4 °C), transferred to 1.5 mL microtubes and stored at −80 °C until further analyses. The tissues were assessed for: superoxide dismutase (SOD) determined following the method described by Sun et al. [3], catalase (CAT) measured following Johansson and Borg [4], glutathione S-transferase (GST) was determined according to Habig et al. [5], and total antioxidant capacity (TAC) levels were determined as described in Madeira et al. [6]. The fish assays were approved by the competent national authorities (DGAV). Statistics were performed using the non-parametric Kruskal–Wallis test to compare differences between exposed and control fish, with a significance level of 5%, using the software Statistica 8.0 (USA).

**Results:**

The highest SOD, GST and CAT activities were found in fish samples, after 14 days of exposure to 100 µg NPs/L. Likewise, the highest TAC levels were determined in samples of fish exposed to 100 µg NPs/L, after 14 days of exposure *via* food. The statistical results showed no significant differences (*p* > .05) between the controls and the fish exposed for 7 days to 50 and 100 µg NPs/L, for all biomarkers. However, significant differences (*p* < .05) were detected between controls and fish exposed for 14 days to 100 µg NPs/L, for all biomarkers analysed.

**Discussion and conclusions:**

Preliminary results show that exposure *via* ingestion of 50 µg NPs/L did not cause significant effects on fish during the experimental period (7 and 14 days), while fish exposed to 100 µg NPs/L showed a significant increase in enzyme activities (SOD, GST, CAT) and TAC levels, after 14 days of exposure suggesting that this concentration of NPs may cause oxidative stress in fish. In addition, the variability found in some results may be due to fish not ingesting the same amount of food containing NPs, as they compete for food. Overall, the present study contributes to a better understanding of the risk of exposure to NPs for aquatic biota.
